# Screening of Plant-Derived Lactic Acid Bacteria for Faba Bean Fermentation and Their Mycotoxin Removal Capacity

**DOI:** 10.3390/microorganisms14061358

**Published:** 2026-06-17

**Authors:** Hang Xiao, Kristóf Kajdi, Reinhard Wimmer, Claus Heiner Bang-Berthelsen

**Affiliations:** 1 Research Group for Microbial Biotechnology and Biorefining, National Institute for Food, Technical University Denmark, Kemitorvet, Building 202, 2800 Kongens Lyngby, Denmark; haxi@food.dtu.dk; 2 Department of Chemistry and Bioscience, Aalborg University, Fredrik Bajers Vej 7H, 9220 Aalborg, Denmark; krka@bio.aau.dk (K.K.); rw@bio.aau.dk (R.W.)

**Keywords:** plant-based foods, faba bean, fermentation, lactic acid bacteria, mycotoxins

## Abstract

The development of novel plant-based products using unconventional food matrices increases the risk of introducing mycotoxins into the food system. Biological detoxification methods, particularly those involving lactic acid bacteria (LAB), are considered sustainable and safe strategies. In this study, we screened 142 plant-derived LAB strains across 17 species for their fermentation performance and mycotoxin removal capacity during faba fermentation. Among them, 84 strains showed rapid acidification. The plating of 11 selected strains confirmed robust growth with cell densities ranging from 4 × 10^8^ to 2.18 × 10^9^ CFU/mL. Screening for aflatoxin B1 (AFB1) removal in complex medium identified several strains that could reduce AFB1 in the supernatant. However, complete toxin extraction after faba fermentation indicated that AFB1 was not enzymatically degraded. Similarly, no significant degradation of ochratoxin A or zearalenone was observed during faba fermentation. Additionally, a cell binding test with 11 selected strains showed that all strains bound AFB1, with efficiencies from about 10% to 35%. Notably, *Lentilactobacillus hilgardii* NFICC857 demonstrated the highest binding capacity, which has never been reported before. Our study provides preliminary insight into plant-derived LAB in mycotoxin removal. Given the vast unexplored diversity of LAB in nature, the discovery of novel strains with enhanced mycotoxin-binding capacity and potential enzymatic degradation remains promising.

## 1. Introduction

The shift toward a plant-based diet has become increasingly popular in recent years, driven by concerns about health, environmental sustainability, and animal welfare. This has led to rapid innovation in developing novel plant-based or hybrid foods with adapted processing technologies [1,2]. As a part of this trend, there is a pressing demand for the complete or partial replacement of animal-derived proteins with plant-based alternatives [3].

Faba bean (*Vicia faba*), also called broad bean, has gained considerable attention due to its agronomic and nutritional advantages [4]. This is because faba bean is a sustainable legume crop that can be easily adapted to a wide range of climates worldwide. In addition, faba bean is rich in protein, dietary fiber, and micronutrients and is not classified as a regulated allergen, making it a promising candidate for developing innovative plant-based food products, such as plant-based cheese and meat alternatives [5].

However, creating novel food matrices by replacing animal-derived ingredients with plant-based components may introduce emerging food safety risks that are not yet fully understood [6]. One of the concerns is the risk of mycotoxin contamination originating from plant materials, as it may occur during crop growth, post-harvest handling, and inappropriate storage [7]. Mycotoxins are toxic secondary metabolites produced by filamentous fungi that can cause both acute and chronic health issues. Along with the increasing demand for pulses, such as faba bean, chickpea, lentil, pea, and common bean, mycotoxin contamination in these commodities has been continuously reported worldwide. Among the various detected mycotoxins, aflatoxins are the most frequently reported contaminants [8,9]. Aflatoxins are produced mainly by *Aspergillus flavus*, *Aspergillus parasiticus*, and *Aspergillus nominus*, with aflatoxin B1 (AFB1) being the most prevalent and toxic form. In addition to aflatoxins, ochratoxin A (OTA) is another mycotoxin commonly detected in pulses. OTA is produced by *Aspergillus* and *Penicillium* and carries a wide range of toxicities and usually enters food stocks during storage, particularly when stored under insufficiently dry conditions [10,11]. Another common mycotoxin is zearalenone (ZEA), a pseudoestrogen produced by a wide range of *Fusarium* species, which are common plant pathogens causing various plant diseases. ZEA often co-occurs with other *Fusarium*-derived mycotoxins, e.g., deoxynivalenol [12]. The presence of mycotoxins in the food chain poses serious public health risks, resulting in significant food waste and substantial economic losses [13].

To reduce the effects of already manifested mycotoxin contamination, various physical and chemical methods such as milling, heating, irradiation, ammonia and ozonization treatment have been extensively studied. However, many of these approaches present critical limitations, including incomplete decontamination, high costs, negative impacts on nutritional quality, and impaired sensory attributes such as appearance, texture, and taste [10,14,15,16,17]. In contrast, biological detoxification methods have emerged as promising alternatives, as they appear to be more sustainable and safer while preserving the nutritional and sensory quality of food materials [18,19].

In particular, lactic acid bacteria (LAB) have attracted increasing interest for their potential application in mycotoxin removal [20]. LAB are generally recognized as safe (GRAS) microorganisms and are widely used in developing innovative legume-based products to improve their nutritional, textural, and sensory properties [21,22]. Previous studies have demonstrated that LAB can remove AFB1, OTA and ZEA predominantly via adsorption to cell wall components, with limited evidence also suggesting the possibility of enzymatic degradation [23,24,25]. Importantly, industrial fermentation pipelines utilizing LAB are well established, enabling the incorporation of new functional strains without major modifications to existing processing technologies. From a practical and industrial perspective, the identification of LAB strains capable of even partial mycotoxin removal represents a realistic, scalable, and sustainable strategy to enhance the safety of faba bean-based and other plant-based food products. So far, the strains noted for efficient aflatoxin removal, primarily through cell surface binding are mostly dairy- or intestinal-derived, such as *Lacticaseibacillus rhamnosus* (*L. rhamnosus*), *Lactobacillus acidophilus* (*L. acidophilus*), *Lacticaseibacillus casei* (*L. casei*) and *Limosilactobacillus reuteri* (*L. reuteri*), which were not fully tested for fermenting complex plant materials [26,27]. Plant-derived LAB are generally considered more adapted to plant fermentation matrices, in light of their better growth and acidification performance in plant substrates [28]. Such adaptation is highly relevant for mycotoxin removal because both mycotoxin binding and potential enzymatic degradation are dependent on sufficient biomass [29].

In this study, we aimed to screen a large set of plant-derived LAB strains for faba bean fermentation by leveraging the DTU National Food Culture Collection (NFICC). In addition, their potential to reduce AFB1, ZEA and OTA levels through degradation and/or binding mechanisms was evaluated. This work seeks to identify LAB strains that are both safe for food applications and effective in mitigating mycotoxin contamination in faba bean-based products, thereby supporting the development of safer and more sustainable plant-based foods.

## 2. Materials and Methods

### 2.1. Strains and Culture Conditions

A total of 142 LAB strains (Table S1), representing 17 species used in this study, were all picked from the National Food Institute culture collection (NFICC). The LAB strains were isolated from plant materials, such as vegetables and fruits from Denmark. All strains belong to the species listed on the European Food Safety Authority (EFSA) Qualified Presumption of Safety (QPS) list. For general cultivation purposes, all LAB strains in this study were cultivated with MLS medium [30] at 30 °C under anaerobic conditions.

### 2.2. High-Throughput Screening for LAB in Faba Fermentation

Faba bean flour (5%, *w*/*v*) derived from Aarhus University was used for screening. Acidification was carried out by using a pH-MultiScan (HNH, Støvring, Denmark) in combination with a pH dye [31]. The pH dye was prepared by using bromocresol green and bromocresol blue (1:1) by following a previously well-defined method [32]. Then 5 mL pH dye was mixed with 95 mL pasteurized (72 °C, 2 min) 5% faba bean flour. A sterile transparent 96-well plate (Corning Inc., Corning, NY, USA) was used for LAB cell cultivation. Overnight precultures were prepared for all strains and adjusted to the same cell density (OD600 = 1.5) prior to inoculation. To ensure sufficient growth and acidification during the screening assay, 1 µL of the preculture was inoculated into 150 µL 5% faba bean flour, without additional sugars. The change in the hue was recorded by pH MultiScan software v6.0.3 every 10 min during 48 h incubation at 30 °C.

A standard curve was established by different pH values in faba bean (adjusted with 1 M lactic acid) with corresponding hues. In parallel, a separate set of fermentations including all strains was performed without the pH dye, and color development was visually evaluated after fermentation. Voges–Proskauer (VP) assay was conducted to evaluate acetoin and diacetyl production. Specifically, 83 μL of fermented faba was transferred to a microplate, followed by the addition of 50 μL of α-naphthol and 16 μL of 40% potassium hydroxide. After incubation in a fume hood for 30 min, red color development was visually evaluated.

### 2.3. Preparation of Medium and Buffer Solution Containing Mycotoxins

AFB1 stock solution (10 mg/mL) was prepared by dissolving AFB1 powder (Sigma-Aldrich, St. Louis, MO, USA) in benzene–acetonitrile (97:3). The AFB1 stock solution was diluted in either culture medium or PBS buffer on the day of use, to achieve appropriate working concentrations (either 2500 ppb or 100 ppb, with 1 ppb defined as 1 ng/g), and the diluted stocks were placed on a heating plate at 80 °C for 10 min to evaporate benzene and acetonitrile in a fume hood [25]. After it cooled down to room temperature, bacterial cells were inoculated accordingly. For ZEA and OTA, stock solutions of 5 mg/mL ZEA in DMSO and 1 mg/mL OTA in DMSO were prepared. Samples spiked with a final concentration of 2500 ppb ZEA or OTA were prepared by directly adding appropriate volumes of the stock solutions.

### 2.4. Broad Screening for AFB1 Removal

To screen out LAB strains potentially removing AFB1 under growing conditions, 10 µL of precultured LAB was inoculated in 1 mL MLS medium containing 2500 ppb AFB1. After incubation at 30 °C for 48 h, cells were spun down, and the supernatant was collected. To quantify the AFB1 concentration in the supernatant, the collected supernatant was diluted 100-fold in PBS (pH = 7.0). ELISA was carried out by using a Celer AFLA HS kit (Gold Standard Diagnostics, Budapest, Hungary) according to the manufacturer’s instructions with internal standards at concentrations of 0, 0.75, 4, 20, and 50 ppb. The yellow color development of the ELISA plate was evaluated by measuring absorbance at 450 nm with a plate reader (Tecan Infinite 200 Pro, Tecan, Männedorf, Switzerland).

### 2.5. Evaluation of Mycotoxin Degradation During Faba Fermentation

Since the microbial removal of mycotoxins relies on biomass, to support the better growth of LAB, faba medium, which consists of 5% faba bean flour and 5% faba protein concentrate (FPC; Vertis, Oslo, Norway), was formulated to test mycotoxin removal under growing conditions. Specifically, precultured LAB strains were inoculated to an initial cell density equivalent to OD_600_ = 0.05 in a deep well plate in faba medium spiked with mycotoxins. After incubation at 30 °C for 48 h, the fermented faba medium was well mixed. Then, methanol was added to a final concentration of 70%, followed by vigorous vortexing for 5 min. The mixture was placed at room temperature for 2 h to fully extract AFB1 from the fermented faba medium. The residual AFB1 after fermentation was evaluated using ELISA as previously described, and residual OTA and ZEA were evaluated by LC-MS.

### 2.6. Characterization of Acidification Using ICinac

iCinac (AMSAlliance, Rome, Italy) was employed to characterize the acidification rate of LAB strains during faba bean fermentation. Precultured LAB cells were inoculated at an initial OD_600_ of 0.1 into faba medium without additional sugars. Calibrated pH probes were placed in 200 mL bottles containing 150 mL of faba medium, and the cultures were incubated at 30 °C. After 21 h of fermentation, when all samples had reached a stable pH, fermentation was stopped by placing the samples on wet ice. Samples were subsequently diluted 10^6^ and 10^7^ times in cold PBS and plated on MLS agar plates for cell enumeration.

### 2.7. Prescreening for OTA and ZEA Removal

The fermentation medium consisted of 5% (*w*/*v*) faba bean powder and 5% (*w*/*v*) faba bean protein suspended in ELGA-grade water. The medium was heat-treated at 80 °C for sanitation and subsequently cooled to room temperature. After cooling, ochratoxin A or zearalenone was added to a final concentration of 2500 ppb. Overnight precultures were prepared for all strains. After washing with saline solution, cell density was assessed by measuring the optical density at 600 nm. The spiked media were inoculated to achieve a final cell density equivalent to OD600 = 0.1 and incubated at 30 °C for 48 h. Following fermentation, the samples were subjected to extraction using a solution of 70% methanol and 4% NaCl. A volume of 5 mL of extraction solvent was added per gram of fermented material (wet weight). The samples were then centrifuged, and the supernatants were collected for analysis. Mycotoxin concentrations in the collected supernatants were determined using enzyme-linked immunosorbent assay (ELISA) kits (Celer OCHRA and Celer ZEA; Gold Standard Diagnostics, Budapest, Hungary), following the manufacturer’s instructions. The absorbance of the developed yellow color was measured at 450 nm using a microplate reader (Tecan Spark, Männedorf, Switzerland).

### 2.8. HPLC-HRMS for OTA and ZEA Quantification

Analytical HPLC-HRMS was performed on a Hitachi LaChrom Elite HPLC system using a C6-phenyl (Kinetex 5 µm 100 Å, 150 × 4.6 mm, Phenomenex, Torrance, CA, USA) column coupled to a high-resolution mass spectrometer (compact qTOF, Bruker, Bremen, Germany) with an electrospray source (Capillary: 4500 V; end plate offset 500 V; Dry gas 4.0 L/min, 200 °C) in positive mode using a 3:97 flowsplitter. The flow was kept constant at 1.2 mL/min with solvent A water (HiPerSolv, VWR, Radnor, PA, USA) and solvent B (100% acetonitrile), both supplemented with 0.1% formic acid. Gradient elution started at 45% solvent B, and the content of solvent B was increased linearly to 60% over 5 min, whereafter it was increased to 100% and kept at 100% B for 4 min. A pre-run period of 6 min was used to flush the column with starting solvent conditions prior to each run.

The injection volume was 40 µL. A standard curve of zearalenone and ochratoxin A was prepared by diluting zearalenone and ochratoxin to 5000, 2500, 1000, 500, 250 and 100 ppb. The standard samples were analyzed in duplicates. Extracted ion chromatograms were prepared capturing the main adducts and fragments of zearalenone (341.14, 319.16, 301.15, 283.13, all ± 0.01 *m*/*z*) and ochratoxin A (426.07, 404.0926, 358.09, 428.07, 406.09, 360.08, all ± 0.01 *m*/*z*) and integrated in BRUKER Compass Data Analysis 4.2. Integrals were converted to concentrations using the standard curves, as demonstrated in the supplementary data.

### 2.9. AFB1 Binding Test Using Resting Cells

Cell binding assay was carried out by using resting cells in PBS buffer, which is a commonly used method for evaluating mycotoxin adsorption by LAB, enabling better comparison with previously published studies. This method follows a previously described method with some small modifications [25]. Precultured LAB cells were spun down and washed twice with PBS, and then cells were resuspended in 1 mL PBS containing 2500 ppb AFB1 at OD_600_ = 10. After incubation at 30 °C for 24 h, samples were centrifuged to fractionate the cell and supernatant. After washing with fresh PBS once, cells were extracted with 70% methanol, followed by vigorous vortexing, and left for 2 h at 25 °C. The AFB1 concentration in the cell fraction and supernatant fraction were evaluated with ELISA as previously described.

### 2.10. Statistical Analysis

Statistical analyses were performed using Origin 2023 software. Data were calculated as the mean ± standard deviation (SD) from two independent measurements. To evaluate the removal of AFB1, OTA, and ZEA during faba bean fermentation, an independent Student’s *t*-test was applied to compare mycotoxin levels in fermented samples with the non-fermented control. Differences were considered statistically significant if the *p*-value was lower than 0.05.

## 3. Results and Discussion

### 3.1. Screening LAB Strains in Efficient Faba Bean Fermentation

**Figure 1:**
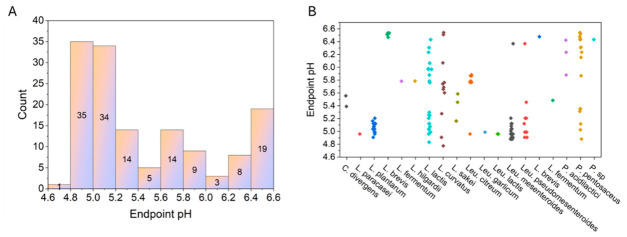
Strain screening for efficient fermentation in faba bean. A total of 142 plant-derived LAB strains were characterized with acidification during faba fermentation using pH dye. (**A**) The distribution of endpoint pH for 142 LAB strains after 24 h of fermentation in faba bean. The count of strains is indicated in each bin. (**B**) The distribution of endpoint pH among LAB species, which are indicated with different colors.

To screen out LAB strains potentially used for AFB1 removal from faba, a total of 142 plant-derived LAB strains across 17 species were evaluated for faba bean fermentation without providing additional sugars. Fermentation efficiency was assessed by the acidification profile, which is commonly associated with LAB growth. As shown in Figure 1A, 112 strains reached a pH value lower than 6.0 during 24 h, 84 strains reached pH 5.4, 70 strains reached pH 5.2, and 36 strains reached pH 5.0. This indicated excellent fermentation performance (pH < 5.4) by most of the tested LAB strains in faba bean. Notably, the endpoint pH showed a species-dependent pattern (Figure 1B). We defined an endpoint pH of less than 5.4 after 24 h as efficient acidification. These 84 strains include 12 LAB species, containing all 18 tested *Lactiplantibacillus plantarum* (*L. plantarum*), most *Leuconostoc* strains, approximately half of *Lactococcus lactis* (*L. lactis*) and one-third of *Latilactobacillus curvatus* (*L. curvatus*) and *Pediococcus pentosaceus* (*P. pentosaceus*), as well as 1 *Carnobacterium divergens* (*C. divergens*), 2 *Latilactobacillus sakei* (*L. sakei*) and 1 *Lacticaseibacillus paracasei* (*L. paracasei*). These results highlighted the decent fermentability of faba bean by *L. plantarum* and *Leuconostoc* spp. One exception is *Leuconostoc citreum* (*Leu. citreum*), where only one out of nine strains was shown to be efficient in acidification during screening. Slow acidification generally indicates limited metabolic activity and the weaker growth of LAB in fermenting faba, which reduces the likelihood of effective mycotoxin. A total of 30 strains showing slow acidification were excluded from subsequent AFB1 detoxification studies.

*L. plantarum* and *Leuconostoc mesenteroides* (*Leu. mesenteroides*) are typical LAB species commonly found in plant niches and are among the most frequently isolated species from diverse plant materials [30]. Their strong fermentation performance in this study further demonstrates their broad metabolic capacity toward complex plant substrates such as faba bean. From a practical aspect, a rapid acidification rate and low endpoint pH are favorable for controlling the growth of undesirable microorganisms and extending the storage life of fermented products. Importantly, rapid acidification usually indicates efficient cell growth, which is critical for effective AFB1 removal, via either potential cell binding or enzymatic degradation mechanisms [33,34]. Beyond acidification capacity, *L. plantarum* and *Leuconostoc* spp. are frequently noted for their excellent ability to reduce plant-derived off-flavors and antinutritional factors, which may provide additional advantages for plant-based fermentation [35,36].

### 3.2. Color and VP Characterization After Faba Bean Fermentation

During faba bean fermentation screening, distinct color development was observed. A total of 64 strains produced a white gel, whereas others retained the natural dark color, which was identical to the color of the unfermented faba bean (Table S2). Furthermore, *Levilactobacillus brevis* (*L. brevis*), *L. lactis*, and *Leu. citreum* frequently exhibited a white color after fermentation. In contrast, neither *L. curvatus* nor *L. sakei* exhibited white color development and retained the original dark color. Similarly, the VP assay results also showed a strong correlation with LAB species. In total, 58 strains were characterized as VP-positive in faba bean fermentation. Interestingly, *L. curvatus*, *L. plantarum*, *L. lactis*, and *P. pentosaceus* represent the majority of VP-positive strains. In contrast, only a few strains of *L. brevis*, *Leu mesenteroides*, *L. sakei*, *Leuconostoc pseudomesenteroides* (*Leu. pseudomesenteroides*) and *Leu. citreum* were characterized as VP-positive.

The presence of phenolic compounds and their oxidative reactions is widely found to cause browning in legumes [37]. Faba bean is rich in phenolic compounds, including a large amount of tannins, which contribute to its dark color [38]. The potential to reduce dark color through fermentation would be valuable when formulating plant-based products, such as dairy alternatives, where a light color is usually desirable. In line with the present observations, Kantanen et al. reported that LAB-fermented faba bean exhibited a lighter color, which was due to the degradation of phenolic compounds and low pH after fermentation [39]. In the current study, there was no strong correlation between final pH and color development across the tested strains. This indicated that pH alone is insufficient to explain the observed whitening effect. Given that LAB are able to degrade phenolic compounds, including tannins, through enzymatic activities, the reduced dark color after fermentation might suggest a decreased level of phenolic compounds.

The Voges–Proskauer (VP) assay was used to assess the production of buttery aroma compounds, namely acetoin and diacetyl [40]. This is usually favorable for the production of various foods. More importantly, diacetyl was recognized as an anti-fungal agent that could efficiently inhibit the growth of *Aspergilli* [41,42]. This highlights the potential use of VP-positive strains for aflatoxin control. Similarly to the observations for white color development, the VP results were not correlated with acidification but strongly correlated with LAB species. Our previous work demonstrated that the production of buttery aroma compounds is influenced not only by microbial species but also by the type of plant-based substrate used for fermentation [30]. Consistent with these findings, the present study indicates that strains such as *L. curvatus*, *L. plantarum*, *L. lactis*, and *P. pentosaceus* are more likely to produce butter aroma compounds in faba bean fermentation.

### 3.3. Screening LAB Strains for AFB1 Removal

**Figure 2:**
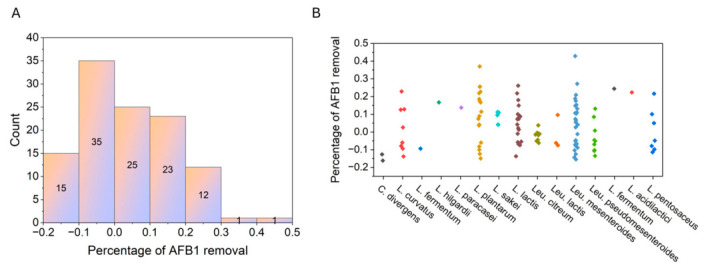
Screening for potential AFB1 removal LAB strains. (**A**) The distribution of the AFB1 removal ratio for 112 LAB strains. The count of strains is indicated in each bin. The AFB1 removal ratio is defined as *C*_s_/*C*_c_, where *C*_s_ is the residual AFB1 concentration in the supernatant, and *C*_c_ is the tested AFB1 concentration in the control. (**B**) The distribution of the AFB1 removal ratio against LAB species, which are indicated with different colors.

After prescreening for faba bean growth, we selected 112 strains for AFB1 removal. To assess their AFB1 removal capacity under growth conditions, all 112 LAB strains were screened in MLS medium spiked with 2500 ppb AFB1. After 48 h of fermentation at 30 °C, the concentration of the remaining AFB1 in the supernatant was investigated. As shown in Figure 2, 62 strains showed AFB1 removal compared to the control, and 37 strains showed more than 10% AFB1 removal; 14 strains showed more than 20% removal. In addition, no strong correlation was observed between AFB1 removal and LAB species.

Similarly, one recent study characterized a total of 31 plant-derived LAB strains including *Weissella cibaria* (*W. cibaria*), *L. pseudomesenteriodes*, *L. plantarum*, *Lactobacillus helveticus* (*L. helveticus*), and *L. citreum* toward AFB1 removal in MRS, and it showed that all strains exhibited an AFB1 binding ratio up to 10% within 24 h incubation, and this nearly doubled when incubation was prolonged to 48 h [43]. In this study, since we only measured the AFB1 left in the supernatant during screening, the reduction in the AFB1 level was potentially due to enzymatic degradation or cell surface binding or both. A total of 11 top-performing strains identified during screening were selected for the further investigation of AFB1 removal in faba bean fermentation.

### 3.4. Acidification Rate and Cell Growth in Faba Bean

**Figure 3:**
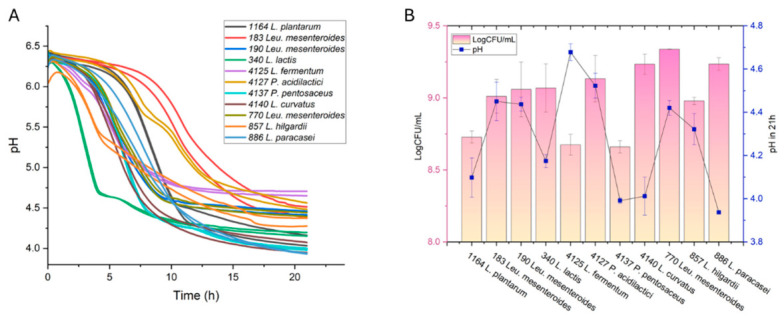
Evaluation of acidification and growth of 11 plant-derived LAB in fermenting faba medium. (**A**) Acidification profile at 30 °C. Strains with biological replicates are indicated with different colors. (**B**) After 21 h of growth, CFU was counted, and endpoint pH is indicated by blue lines. All strains were assessed in biological duplicates.

To confirm the growth and acidification profiles of 11 selected strains, a matrix consisting of 5% faba bean flour and 5% FPC was used; hereafter, we call it faba medium. LAB strains were inoculated into faba medium at an OD600 of 0.1. As shown in Figure 3A, all strains reached pH 5 within 15 h, with NFICC 340 *Lactococcus lactis* showing the fastest acidification, reaching pH 5 within 4 h. In contrast, NFICC183 *Leu. mesenteroides*, NFICC4127 *Pediococcus acidilactici* (*P. acidilactici*), and NFICC1164 *L. plantarum* showed a longer lag phase on acidification than other strains. After 21 h of fermentation in broth, cells were plated to investigate growth by counting CFU. As shown in Figure 3B, all strains exceeded 4 × 10^8^ CFU/mL, with more than half exceeding 10^9^ CFU/mL and NFICC 770 *Leu. mesenteroides* achieving 2.18 × 10^9^ CFU/mL. NFICC 340, the fastest acidifying strain, reached 1.22 × 10^9^ CFU/mL.

LAB growth can be influenced by the buffering capacity of the medium, as higher buffering capacity can enhance biomass production, which is crucial for AFB1 removal [29]. In this study, we compared the buffering capacity of different matrices, including soy as a reference (Figure S1). Among the tested samples, 5% faba bean flour exhibited the lowest buffering capacity. In contrast, 5% FPC showed a buffering capacity comparable to that of 5% soy flour. A mixture of 5% faba bean flour + 5% FPC achieved nearly three times the buffering capacity of 5% faba bean flour alone. In addition, CFU and endpoint pH are not strongly correlated, likely because of the differences in acid production and tolerance among LAB strains. Overall, the results confirmed the robust growth and acidification of the tested LAB strains in faba fermentation even without the addition of sugars.

### 3.5. AFB1 Removal During Faba Bean Fermentation

**Figure 4:**
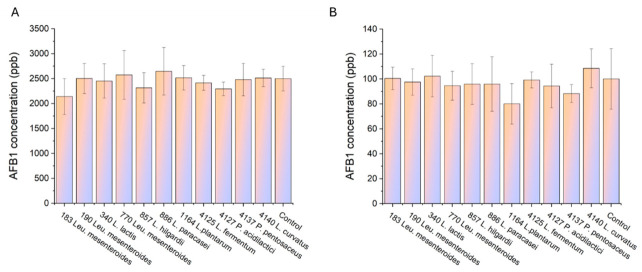
Assessment of AFB1 detoxification of 11 strains in fermenting faba medium. Complete extraction was conducted to determine total AFB1 left after 48 h fermentation of faba medium spiked with (**A**) 2500 ppb AFB1 and (**B**) 100 ppb AFB1. All strains were assessed in biological duplicates.

To assess the strains in terms of AFB1 removal during fermentation, LAB strains were inoculated into faba medium spiked with either 2500 ppb or 100 ppb AFB1. After 48 h, total aflatoxin extraction was performed on the entire fermentation product to assess AFB1 degradation. As shown in Figure 4A, all the tested strains exhibited a similar AFB1 concentration compared to the control. Although NFICC183 *Leu. mesenteroides*, NFICC857 *Lentilactobacillus hilgardii* (*L. hilgardii*), and NFICC4127 *P. acidilactici* showed slight AFB1 reductions when spiked with 2500 ppb AFB1, these reductions were not statistically significant. Similarly, as shown in Figure 4B, when spiked with 100 ppb, NFICC1164 *L. plantarum* and NFICC4137 *P. pentosaceus* showed a slight reduction; however, no statistical difference was observed. Overall, no significant reduction in AFB1 levels was detected for any of the tested strains, indicating that none of the characterized LAB strains were able to degrade AFB1 during fermentation.

LAB occur naturally in spontaneous food fermentations and are widely used as starter cultures in the food and beverage industry [44]. In this study, we attempted to screen out plant-derived LAB capable of biotransforming aflatoxins during faba fermentation; however, none of the strains exhibited clear AFB1 removal after complete AFB1 extraction from the fermented product. One similar study characterized *L. plantarum*, *L. brevis*, and *P. pentosaceus* toward AFB1 removal in faba fermentation, and a reduction of 90% in the supernatant was observed when spiked with only 10 ppb aflatoxin B1 [45]. However, this reduction was reported to be due to the cell binding mechanism rather than biotransformation. This is consistent with the fact that complete biotransformation has been reported scarcely by LAB, since LAB lacks relevant enzymes for AFB1 degradation. Interestingly, one recent study shows that LAB such as *L. paracasei*, *P. acidilactici*, *P. pentosaceus*, *L. plantarum* and *L. curvatus* are able to partially transform B1into Aflatoxicol, aflatoxin P2, aflatoxin Q1 and aflatoxin B2a, which are less toxic than aflatoxin B1 during fermentation [24]. Additionally, some studies showed that LAB could achieve a high AFB1 biotransformation rate when followed by heating processes. Chen et al. showed that *Streptococcus thermophilus* and *Lactobacillus delbrueckii* could biotransform AFB1 by introducing a heating step at 100 °C, though the removal mechanisms and end products are not fully understood yet [23]. Another study shows that heating AF with lactic acid (pH 3–4) at a moderately high temperature, 80 °C, shows 85% degradation of the aflatoxins within 2 h [46]. This indicates that certain LAB strains have the potential to facilitate AFB1 biotransformation under specific conditions, such as low pH and when combined with the heating process.

In contrast to LAB, the enzymatic degradation of aflatoxins has been more frequently reported in other microorganisms such as *Bacillaceae*, *Pseudomonadaceae*, *Myxomycophyta*, *Flavobacteriaceae*, *Nocardiaceae*, *Candida* spp., *Aspergillus* spp., *and Rhizopus* spp. [15,44]. Although effective AF removal can be achieved using such unconventional microorganisms, critical safety and quality concerns must be addressed before they can be used broadly. One concern is that the metabolites derived from the incomplete degradation of aflatoxins may retain toxicity. In addition, many of those non-traditional bacteria or fungi are often isolated from soil or other environmental sources rather than being GRAS, which may pose risks due to the production of hazardous compounds or undesirable flavors and tastes. Furthermore, these unconventional strains may outcompete beneficial microflora, resulting in spoilage. Such challenges complicate their integration into existing food processing pipelines and facilities, thereby limiting their practical applications.

### 3.6. OTA and ZEA Removal During Faba Fermentation

In addition to AFB1, the potential of OTA and ZEA removal during faba fermentation was also examined. Since OTA and ZEA removal may not be associated with the AFB1 results, 29 strains (Table S3) were independently picked based on their acidification during the initial screening. OTA and ZEA analyses were first conducted using ELISA after complete toxin extraction from the fermented matrix. The results showed no significant OTA or ZEA removal compared with the control samples (Figures S2 and S3). However, considering the complexity of the faba bean fermentation matrix, where ELISA measurements may be affected by matrix interference or cross-reactivity, 16 representative strains were subsequently selected for further validation by HPLC-HRMS analysis. These included strains showing the lowest residual OTA and ZEA concentrations as well as strains with comparatively high residual toxin levels. Figure S4 displays the excellent linearity of the standard series of ZEA and OTA. An example chromatogram with the extracted ion chromatograms and mass spectra of ZEA and OTA is shown in Figure S5.

**Figure 5:**
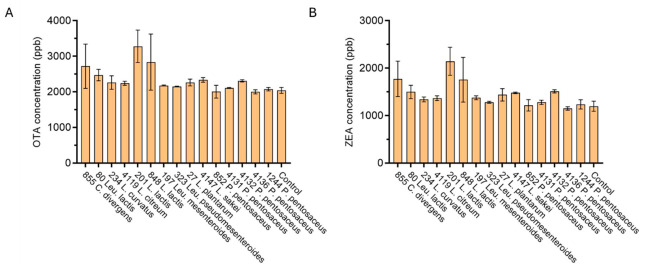
Assessment of OTA and ZEA detoxification in fermenting faba medium. Complete extraction was conducted to determine total OTA (**A**) and ZEA (**B**) left after 48 h of fermentation in faba medium. All strains were assessed in biological duplicates.

After 48 h of fermentation in faba medium, complete OTA and ZEA extraction was performed, and the remaining OTA and ZEA after faba fermentation were investigated by HPLC-HRMS. As shown in Figure 5, both ZEA and OTA were detected in all samples after fermentation. However, no significant reduction in OTA or ZEA was observed in any of the tested strains compared to the unfermented control. These results validated that none of the investigated LAB strains were able to effectively degrade OTA or ZEA during faba fermentation. These HPLC-HRMS results were consistent with the initial ELISA screening results, confirming the reliability of the ELISA and demonstrating that none of the investigated LAB strains were able to effectively degrade OTA or ZEA during faba fermentation.

Previous studies have reported the enzymatic degradation of OTA and ZEA using certain LAB strains; however, degradation is often found to be incomplete [47,48]. Similarly to AFB1, the predominant mechanism reported for OTA and ZEA removal by LAB is still adsorption to the bacterial cell wall rather than enzymatic degradation [49,50,51]. This partially explains why, in this study, no OTA or ZEA reduction has been observed after complete toxin extraction from the fermented products. A recent study suggested that laccases derived from LAB may be involved in OTA degradation, and their activity can be enhanced by the presence of (-)-epicatechin and copper, providing a novel approach for OTA breakdown [52]. For ZEA, certain transformation pathways may lead to the formation of metabolites with increased estrogenic derivatives, such as trihydroxy zearalenol and trihydroxy zearalenol sulfate; however, the underlying mechanisms in LAB remain poorly characterized [53].

In the present study, no detectable reduction in OTA or ZEA was observed after complete toxin extraction. This indicates that the tested strains were ineffective in enzymatically degrading OTA and ZEA during faba fermentation. It remains unclear whether this outcome reflects an intrinsic inability of the selected LAB strains to degrade these mycotoxins or whether potential degradation was suppressed by the faba bean matrix itself.

### 3.7. AFB1 Cell Binding Assay

**Figure 6:**
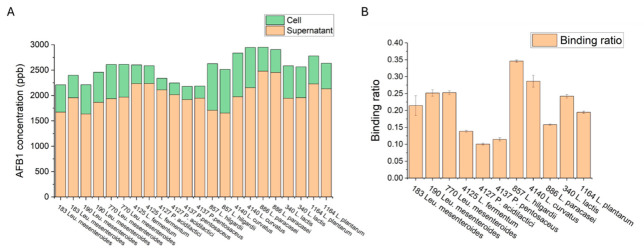
Characterization of AFB1 binding efficiency for different LAB strains. Resting cells (OD_600_ = 10) were inoculated into PBS spiked with 2500 ppb AFB1. (**A**) After 24 h of incubation, AFB1 was quantified in both cell (green) and supernatant (yellow) fractions. (**B**) AFB1 cell binding ratio relative to total AFB1 recovered from supernatant and cell fractions; AFB1 binding ratio was defined as *C*_cell_/(*C*_cell_ + *C*_supnatant_). All strains were characterized by biological duplicates.

To assess the binding efficiency of different strains towards AFB1, a binding assay was conducted using resting cells (OD_600_ = 10) in PBS spiked with 2500 ppb AFB1. After 24 h of incubation at 30 °C, the cells were separated from the supernatant, and AFB1 was quantified in both fractions. As shown in Figure 6A, all tested strains were able to bind AFB1, and the biological duplicates exhibited similar patterns, indicating good reproducibility. Figure 6B further shows that all strains achieved at least 10% AFB1 binding, while NFICC-857 *L. hilgardii* exhibited the highest binding ratio of 35%, followed by NFICC-4140 *L. curvatus* 29%. Moreover, *Leu. mesenteroides* NFICC-190, NFICC-770, and *L. lactis* NFICC-340 showed around a 25% binding ratio, and *Leu. mesenteroides* NFICC-183 and *L. plantarum* NFICC-1164 showed around 20% binding.

Although LAB lack the necessary enzymes for effective aflatoxin degradation, cell binding remains a promising approach for aflatoxin removal. Many studies have demonstrated that LAB can remove aflatoxin through cell wall binding mechanisms. AFB1 cell binding assays are usually carried out with cells suspended in a buffer solution containing aflatoxins. Therefore, decreased AFB1 levels in such conditions is more likely due to cell binding mechanisms rather than enzymatic reactions. However, specific conditions, such as incubation duration, cell density, buffer composition, pH, temperature, aflatoxin concentration, and cell pretreatment, may vary. These variations must be carefully considered when comparing results across different studies. Nevertheless, the most documented strains for efficient aflatoxin binding are *L. rhamnosus*, *L. casei*, *Streptococcus thermophilus*, *L. acidophilus*, and *L. reuteri* [25,27,54]. This implies that these species are more likely to exhibit strong AFB1 binding capacity. It is important to note that these LAB species known for efficient aflatoxin binding are mostly derived from gastrointestinal or dairy sources. Despite their strong cell binding capabilities toward AFB1, their effectiveness in fermenting various plant-based materials is still questionable. In addition, an ideal model for mycotoxin removal would involve minimal adhesion to the intestinal track and rapid elimination from the body [44]. In this study, we tested several plant-derived LAB species for AFB1 binding, such as *Leu. mesenteroides*, *L. fermentum*, *Pediococcus acidilactici*, *Pediococcus pentosaceus*, *L. hilgardii*, *L. curvatus*, *L. paracasei*, *L. lactis*, and *L. plantarum*. None of them achieved an AFB1 binding efficiency comparable to that of strains such as *L. rhamnosus* GG under similar experimental conditions [25,26]. Interestingly, *L. hilgardii* showed a binding efficiency of 35%, which is the highest value among the strains we tested. To the best of our knowledge, this is the first time *L. hilgardii* has been reported for AF removal. The other strains we tested have binding efficiencies ranging from 10% to 29%. Similar observations were also observed by characterizing AFB1 binding using plant-derived strains in recent studies: Jenna Lemmetty characterized 31 LAB, including plant-derived species *Leu. citreum*, *Leu. pseudomesenteroides*, *L. plantarum*, *P. pentosaceus*, *W. cibaria* and *W. confuse*, and showed up to 41% binding efficiency across all strains using 75 mg variable cells in 1.5 mL buffer containing 1000 ppb AFB1 [43]. Sarra Rafai evaluated 10 strains for AFB1 binding including *L. paracasei*, *P. acidilactici*, *P. pentosaceus*, *L. plantarum*, and *L. curvatus*, showing up to only 5% AFB1 binding efficiency in 200 ppb conditions [24].

LAB are Gram-positive bacteria with a thick, complex cell wall, which plays a pivotal role in AFB1 binding. Early studies showed that cell-bound AFB1 can be largely recovered with organic solvents, such as methanol, chloroform and benzene, which indicates the binding is noncovalent and reversible [25]. During this process, cell wall components such as peptidoglycan, teichoic acids, and polysaccharides all contribute to AFB1 sequestration. However, the effects are highly strain-specific. For example, teichoic acids were demonstrated to play a key role in binding AFB1 in *L. casei* Shirota but not in *L. rhamnosus* GG. This was presumably because the different composition and substructure of the teichoic acid components alters the surface hydrophobic and electrostatic properties, which is crucial for AFB1 binding efficiency [26,55,56]. In this sense, cell wall-associated proteins may also interfere with AFB1 binding, even though they are unlikely to bind to AFB1 directly. The S-layer protein, which acts as an outer shell of the LAB cell surface, was usually considered a barrier to the accessibility of AFB1 to peptidoglycan [25,45,57]. One piece of evidence is that *L. rhamnosus* GG does not possess a thick S-layer protein, which might further facilitate its AFB1 binding capability [25]. Despite these findings, the cell binding mechanisms toward AFB1 are still not fully understood in LAB. Given the structural diversity among LAB species [58] and the vast unexplored LAB in nature, the discovery of novel strains with enhanced mycotoxin binding capacity remains a promising research direction.

## 4. Conclusions

This study screened 142 plant-derived LAB strains for their ability to acidify and remove mycotoxins during fermentation. A total of 84 strains showed rapid acidification, indicating their robust growth in faba fermentation. This was further confirmed by 11 selected strains which reached cell densities above 4 × 10^8^ CFU/mL after faba fermentation, while several strains exceeded 10^9^ CFU/mL.

This study further investigated the potential removal of AFB1, OTA, and ZEA during faba fermentation. However, none of the tested strains showed significant irreversible removal of the investigated mycotoxins after complete toxin extraction from the fermented matrix. In the case of AFB1, several strains showed positive binding behavior, suggesting reversible surface absorption rather than true degradation, with *L. hilgardii* showing the highest binding ratio at approximately 35%. In contrast, OTA and ZEA binding mechanisms were not separately investigated.

Future studies should focus on optimizing fermentation conditions, such as pH and temperature, and further investigating reversible adsorption mechanisms to better understand LAB–mycotoxin interactions.

## Data Availability

The original contributions presented in this study are included in the article/Supplementary Material. Further inquiries can be directed to the corresponding author.

## Supplementary Materials

The following supporting information can be downloaded at https://www.mdpi.com/article/10.3390/microorganisms14061358/s1, Table S1. The strains used in this study; Table S2. Color assessment and VP test; Table S3. Strains tested for ZEA and OTA removal; Figure S1. Comparison of the buffering capacity of different plant-based matrices. The buffering capacity of each plant-based matrix was measured using 40 mL of sample titrated with 85% lactic acid; Figure S2: OTA concentrations in supernatant after incubation with various microorganisms. The data represent the analytical duplicates of biological duplicates, analyzed by ELISA assay. Strains marked with an asterisk represent the strains that showed either significantly higher or lower values than the control and were selected for further LC-MS based screening; Figure S3: ZEA concentrations in supernatant after incubation with various microorganisms. The data represent the analytical duplicates of biological duplicates, analyzed by ELISA assay. Strains marked with an asterisk represent the strains that showed either significantly higher or lower values than the control and were selected for further LC-MS based screening; Figure S4: LC-MS standard curve for LC-MS based quantification of ZEA (blue) and OTA (red). Standard concentrations were recorded in duplicates; Figure S5: LC-MS chromatogram of supernatant from control incubation of faba bean medium spiked with ZEA and OTA. The upper panel shows the base peak chromatogram (black) and the extracted ion chromatograms for ZEA (magenta) and OTA (cyan). The lower panels show the mass spectra extracted at the retention times of the two compounds. Theoretical m/z values are: ZEA: [M+H]^+^: 319.154, [M+Na]^+^: 341.1359, [M-H_2_O+H]^+^: 301.1434 and [M-2 H_2_O+H]^+^: 283.1329; OTA: [M+H]^+^: 404.0895, [M+Na]^+^: 426.0715 and [M-CO_2_+H]^+^ 358.0846 for the ^35^Cl isotopomers. For the calculation of the extracted ion chromatograms, the m/z values of the ^37^Cl isotopomers of these three species was also included.
