# Supplementary material for: Screening of Plant-Derived Lactic Acid Bacteria for Faba Bean Fermentation and Their Mycotoxin Removal Capacity
Source: Microorganisms. 2026 Jun 17;14(6):1358. doi: 10.3390/microorganisms14061358 (PMC13305944; doi:10.3390/microorganisms14061358)
Supplement: Supplementary file 1 [file microorganisms-14-01358-s001.zip › microorganisms-4319369-supplementary.pdf]

Supplementary Material

Screening of Plant-Derived Lactic Acid Bacteria for Faba Bean Fermentation and Their Mycotoxin Removal Capacity

Hang Xiao [1], Kristóf Kajdi [2], Reinhard Wimmer [2] and Claus Heiner Bang-Berthelsen [1,*]

[1] Research Group for Microbial Biotechnology and Biorefining, National Institute for Food, Technical University Denmark, Kemitorvet, Building 202, 2800 Kongens Lyngby, Denmark; haxi@food.dtu.dk

[2] Department of Chemistry and Bioscience, Aalborg University, Fredrik Bajers Vej 7H, 9220 Aalborg, Denmark; krka@bio.aau.dk (K.K.); rw@bio.aau.dk (R.W.)

* Correspondence: claban@food.dtu.dk

Table S1. The strains used in this study

NFICC No.	Species	Origin
19	Lactiplantibacillus plantarum	garden
27	Lactiplantibacillus plantarum	Sourdough, Lyngby
28	Leuconostoc citreum	Sourdough, Lyngby
29	Levilactobacillus brevis	Sourdough, Lyngby
33	Pediococcus pentosaceus	Sourdough, Lyngby
41	Leuconostoc mesenteroides	Sourdough, Lyngby
46	Pediococcus pentosaceus	Sourdough, Lyngby
51	Leuconostoc mesenteroides	Sourdough, Lyngby
71	Leuconostoc mesenteroides	Rosmosevej, Blovstrød
72	Lactiplantibacillus plantarum	Rosmosevej, Blovstrød
80	Leuconostoc garlicum	Rosmosevej, Blovstrød
83	Leuconostoc lactis	Rosmosevej, Blovstrød
86	Lactococcus lactis	Rosmosevej, Blovstrød
94	Leuconostoc citreum	Rosmosevej, Blovstrød
96	Leuconostoc pseudomesenteroides	Rosmosevej, Blovstrød
115	Leuconostoc mesenteroides	Rosmosevej, Blovstrød
121	Levilactobacillus brevis	Rosmosevej, Blovstrød
128	Lactococcus lactis	Frilandsmuseet
142	Lactococcus lactis	Frilandsmuseet
163	Lactiplantibacillus plantarum	Frilandsmuseet
182	Leuconostoc pseudomesenteroides	Frilandsmuseet
183	Leuconostoc mesenteroides	Frilandsmuseet
185	Leuconostoc mesenteroides	Frilandsmuseet
188	Lactococcus lactis	Frilandsmuseet
190	Leuconostoc mesenteroides	Frilandsmuseet
192	Lactococcus lactis	Frilandsmuseet
195	Leuconostoc mesenteroides	Frilandsmuseet
197	Leuconostoc mesenteroides	Rørvig strand
201	Lactococcus lactis	Rørvig strand
202	Leuconostoc pseudomesenteroides	Rørvig strand
204	Leuconostoc mesenteroides	Frilandsmuseet
205	Levilactobacillus brevis	Rørvig strand
208	Lactococcus lactis	Frilandsmuseet
211	Lactococcus lactis	Frilandsmuseet
213	Leuconostoc mesenteroides	Frilandsmuseet
214	Lactococcus lactis	Frilandsmuseet
215	Lactococcus lactis	Frilandsmuseet
224	Lactococcus lactis	Rørvig strand
225	Leuconostoc mesenteroides	Frilandsmuseet

227	Leuconostoc pseudomesenteroides	Rørvig strand
229	Latilactobacillus curvatus	Frilandsmuseet
230	Latilactobacillus curvatus	Frilandsmuseet
231	Latilactobacillus curvatus	Frilandsmuseet
234	Latilactobacillus curvatus	Frilandsmuseet
236	Latilactobacillus curvatus	Frilandsmuseet
238	Lactococcus lactis	Frilandsmuseet
251	Pediococcus pentosaceus	Robin Sour dough M17 isolat 11
256	Leuconostoc mesenteroides	Robin Sour dough MRS isolat 4
259	Latilactobacillus curvatus	Robin Sour dough MRS isolat 7
272	Leuconostoc mesenteroides	Robin Sour dough MRS isolat 20
275	Pediococcus pentosaceus	Robin Sour dough MRS isolat 23
289	Lactiplantibacillus plantarum	Drangsholm slot
298	Leuconostoc mesenteroides	Drangsholm slot
310	Leuconostoc mesenteroides	DTU Campus
314	Leuconostoc pseudomesenteroides	Frilandsmuseet
318	Leuconostoc mesenteroides	55.843098,12.530417
320	Leuconostoc mesenteroides	Svalegabet 10, 2850 Nærum
321	Leuconostoc mesenteroides	Rævehøjvej, DTU
323	Leuconostoc pseudomesenteroides	Lundtofte vej 131 2800 lyngby
328	Latilactobacillus curvatus	Skanshage, Rørvig
335	Lactococcus lactis	Sour dough, Lyngby
338	Lactococcus lactis	Odense NV
339	Leuconostoc pseudomesenteroides	Odense NV
340	Lactococcus lactis	DTU, brewery
341	Pediococcus pentosaceus	DTU, brewery
514	Carnobacterium divergens	Pligtgårdsvej 19, 2660 Brøndby Strand
629	Lactiplantibacillus plantarum	Rosmosevej, Blovstrød
636	Leuconostoc pseudomesenteroides	Rosmosevej, Blovstrød
761	Lactococcus lactis	43 original strains
770	Leuconostoc mesenteroides	43 original strains
773	Lactococcus lactis	43 original strains
782	Leuconostoc mesenteroides	Kursus efterår 2020
798	Lactiplantibacillus plantarum	Zoo kursus efterår 2019
806	Leuconostoc lactis	Zoo kursus efterår 2019
810	Lactiplantibacillus plantarum	Zoo kursus efterår 2019
815	Pediococcus pentosaceus	Zoo RK&Claus isolation
822	Leuconostoc mesenteroides	Zoo RK&Claus isolation
823	Lactiplantibacillus plantarum	Zoo RK&Claus isolation
825	Leuconostoc mesenteroides	Zoo RK&Claus isolation

829	Leuconostoc citreum	Zoo RK&Claus isolation
830	Latilactobacillus sakei	Zoo RK&Claus isolation
844	Lactococcus lactis	Zoo RK&Claus isolation
848	Lactococcus lactis	Zoo RK&Claus isolation
852	Pediococcus pentosaceus	Lise potato
856	Levilactobacillus brevis	Lise potato
857	Lentilactobacillus hilgardii	Lise potato
862	Lactiplantibacillus plantarum	Lise potato
864	Limosilactobacillus fermentum	Lise potato
867	Lactococcus lactis	Lise potato
883	Lactococcus lactis	Lise potato
884	Leuconostoc mesenteroides	Lise potato
885	Carnobacterium divergens	Lise potato
886	Lacticaseibacillus paracasei	Lise potato
900	Latilactobacillus sakei	Lise potato
901	Lactiplantibacillus plantarum	Lise potato
916	Leuconostoc mesenteroides	Lise potato
987	Latilactobacillus curvatus	Lise potato
1033	Lactococcus lactis	Lise potato
1160	Pediococcus pentosaceus	Zoo RK&Claus isolation
1164	Lactiplantibacillus plantarum	Zoo RK&Claus isolation
1187	Leuconostoc mesenteroides	Zoo RK&Claus isolation
1244	Pediococcus pentosaceus	Zoo kursus efterår 2019
1306	Lactiplantibacillus plantarum	Zoo kursus efterår 2019
1323	Lactiplantibacillus plantarum	Lise potato
1324	Leuconostoc citreum	Lise potato
1331	Leuconostoc mesenteroides	Lise potato
1333	Latilactobacillus sakei	Lise potato
1338	Levilactobacillus brevis	Lise potato
1482	Pediococcus pentosaceus	Kursus forår 2020
1488	Lactococcus lactis	Kursus forår 2020
4119	Leuconostoc citreum	70% wheat 30% graham. Room temp
4120	Leuconostoc mesenteroides	white cabbage
4121	Leuconostoc mesenteroides	pointed cabbage
4122	Leuconostoc pseudomesenteroides	pointed cabbage
4123	Leuconostoc pseudomesenteroides	savoy cabbage
4124	Levilactobacillus brevis	feeded daily in the beginning. 60-80% whole wheat, the rest a mix of whole grain, graham and durum
4125	Limosilactobacillus fermentum	pointed cabbage

4127	Pediococcus acidilactici	50 g of Whole corn grain + 50 g of Wheat flour + 125warm water
4128	Pediococcus acidilactici	green cabbage1.5 kg
4129	Pediococcus acidilactici	pointed cabbage
4130	Pediococcus pentosaceus	Wheat 50%, hole grain wheat 50%, feed and mix dayly, 7 days room tepm, dark place, anarobic conditions! Ubs:)
4131	Pediococcus pentosaceus	Rug: feed and mix every couple of days both in fridge/room temp with rye flour Hvede: feed and mix once a week with wheat flour started with a bit of yeast
4132	Pediococcus pentosaceus	white cabbage
4133	Pediococcus pentosaceus	feeded daily, random mix of wheat, rye flower and organic flour
4134	Pediococcus pentosaceus	feeded daily, random mix of wheat, rye flower and organic flour
4135	Pediococcus pentosaceus	pointed cabbage
4136	Pediococcus pentosaceus	feeded daily in the beginning. 60-80% whole wheat, the rest a mix of whole grain, graham and durum
4137	Pediococcus pentosaceus	Fed daily for ca 14 days, 50/50 wheat and rye flour, room temp, a bit colder for 4 days and then room temp again, no direct sunlight, after 5 days, only 100 g lukewarm water was used
4138	Pediococcus pentosaceus	feeding 7 days 50/50 wheat and whole grain wheat, 100g + 125 g warm water. Kitchen table.
4139	Pediococcus sp	pointed cabbage
4140	Latilactobacillus curvatus	50g wheat flour, 25g rye flour, 25g graham flour all organic
4141	Latilactobacillus curvatus	feeded daily in the beginning. 60-80% whole wheat, the rest a mix of whole grain, graham and durum
4142	Latilactobacillus curvatus	feed and mix daily, 40% wheat, 40% grahamsmel, 20% whole grain wheat
4143	Lactiplantibacillus plantarum	50g wheat flour, 25g rye flour, 25g graham flour all organic
4144	Lactiplantibacillus plantarum	Rug: feed and mix every couple of days both in fridge/room temp with rye flour Hvede: feed and mix once

		a week with wheat flour started with a bit of yeast
4145	Lactiplantibacillus plantarum	pointed cabbage
4146	Lactiplantibacillus plantarum	pointed cabbage
4147	Latilactobacillus sakei	pointed cabbage
4148	Leuconostoc citreum	Fed daily in the beginning, 50 g wheat flour, 25 g rye flour, 25 g tipo 0 flour, room temp without direct sunlight, 1 day in direct sunlight.
4149	Leuconostoc citreum	feeded daily in the beginning. 60-80% whole wheat, the rest a mix of whole grain, graham and durum
4150	Leuconostoc citreum	pointed cabbage
4151	Leuconostoc citreum	pointed cabbage

Table S2. Color assessment and VP test

Species	White color	VP assay (butter aroma production)	pH 24h
Carnobacterium divergens	White	-	5.56
Carnobacterium divergens	Dark	-	5.39
Lacticaseibacillus paracasei	White	+	4.96
Lactiplantibacillus plantarum	White	+	5.12
Lactiplantibacillus plantarum	White	+	5.04
Lactiplantibacillus plantarum	White	+	5.04
Lactiplantibacillus plantarum	Dark	+	5.08
Lactiplantibacillus plantarum	Dark	+	4.91
Lactiplantibacillus plantarum	Dark	+	5.04
Lactiplantibacillus plantarum	Dark	+	5.12
Lactiplantibacillus plantarum	Dark	+	5.12
Lactiplantibacillus plantarum	Dark	+	5.16
Lactiplantibacillus plantarum	White	+	4.96
Lactiplantibacillus plantarum	White	+	4.99
Lactiplantibacillus plantarum	Dark	+	5.08
Lactiplantibacillus plantarum	Dark	+	5.04
Lactiplantibacillus plantarum	Dark	+	4.99
Lactiplantibacillus plantarum	Dark	+	5.04
Lactiplantibacillus plantarum	White	-	5.21
Lactiplantibacillus plantarum	Dark	+	5.08
Lactiplantibacillus plantarum	Dark	+	5.04
Lactobacillus brevis	Dark	-	6.47
Lactobacillus brevis	White	-	6.53

Lactobacillus brevis	White	-	6.54
Lactobacillus brevis	White	-	6.54
Lactobacillus brevis	White	-	6.51
Lactobacillus fermentum	White	-	5.78
Lactobacillus hilgardii	White	-	5.79
Lactococcus lactis	Dark	-	5.04
Lactococcus lactis	Dark	-	5.12
Lactococcus lactis	Dark	+	5.88
Lactococcus lactis	White	+	5.08
Lactococcus lactis	Dark	-	5.97
Lactococcus lactis	White	-	4.83
Lactococcus lactis	Dark	-	5.98
Lactococcus lactis	White	+	5.04
Lactococcus lactis	White	-	5.30
Lactococcus lactis	White	-	6.24
Lactococcus lactis	White	+	5.78
Lactococcus lactis	White	+	6.07
Lactococcus lactis	White	-	6.31
Lactococcus lactis	White	+	6.43
Lactococcus lactis	Dark	+	4.99
Lactococcus lactis	White	+	5.12
Lactococcus lactis	White	+	5.25
Lactococcus lactis	Dark	-	5.21
Lactococcus lactis	White	+	4.96
Lactococcus lactis	Dark	-	5.26
Lactococcus lactis	White	-	5.76
Lactococcus lactis	Dark	+	5.26
Lactococcus lactis	White	-	5.97
Latilactobacillus curvatus	Dark	+	5.74
Latilactobacillus curvatus	Dark	+	5.66
Latilactobacillus curvatus	Dark	+	5.76
Latilactobacillus curvatus	Dark	+	4.91
Latilactobacillus curvatus	Dark	+	6.54
Latilactobacillus curvatus	Dark	+	6.07
Latilactobacillus curvatus	Dark	+	6.51
Latilactobacillus curvatus	Dark	+	5.60
Latilactobacillus curvatus	Dark	+	5.28
Latilactobacillus curvatus	Dark	+	5.69
Latilactobacillus curvatus	Dark	+	4.77
Latilactobacillus sakei	Dark	-	5.16
Latilactobacillus sakei	Dark	-	5.59

Latilactobacillus sakei	Dark	-	5.45
Latilactobacillus sakei	Dark	-	5.16
Leuconostoc citreum	White	-	5.87
Leuconostoc citreum	White	-	5.77
Leuconostoc citreum	White	-	5.88
Leuconostoc citreum	White	-	5.87
Leuconostoc citreum	White	+	4.96
Leuconostoc citreum	White	-	5.78
Leuconostoc citreum	White	-	5.78
Leuconostoc citreum	White	-	5.77
Leuconostoc citreum	White	-	5.77
Leuconostoc garlicum	Dark	-	4.99
Leuconostoc lactis	Dark	-	4.96
Leuconostoc lactis	Dark	-	4.96
Leuconostoc mesenteroides	White	-	4.91
Leuconostoc mesenteroides	Dark	-	5.12
Leuconostoc mesenteroides	White	-	4.91
Leuconostoc mesenteroides	White	-	6.37
Leuconostoc mesenteroides	White	-	4.96
Leuconostoc mesenteroides	Dark	-	4.96
Leuconostoc mesenteroides	Dark	-	4.88
Leuconostoc mesenteroides	Dark	-	4.88
Leuconostoc mesenteroides	Dark	+	4.88
Leuconostoc mesenteroides	Dark	-	4.96
Leuconostoc mesenteroides	Dark	-	5.12
Leuconostoc mesenteroides	Dark	-	5.04
Leuconostoc mesenteroides	White	-	4.96
Leuconostoc mesenteroides	Dark	-	5.21
Leuconostoc mesenteroides	White	-	5.12
Leuconostoc mesenteroides	Dark	-	4.96
Leuconostoc mesenteroides	Dark	-	4.96
Leuconostoc mesenteroides	White	-	5.04
Leuconostoc mesenteroides	White	-	5.00
Leuconostoc mesenteroides	Dark	-	4.96
Leuconostoc mesenteroides	White	-	5.04
Leuconostoc mesenteroides	White	-	5.12
Leuconostoc mesenteroides	White	-	4.88
Leuconostoc mesenteroides	White	-	5.08
Leuconostoc mesenteroides	White	-	5.04
Leuconostoc mesenteroides	White	-	5.04
Leuconostoc mesenteroides	Dark	-	4.96

Leuconostoc mesenteroides	Dark	-	4.91
Leuconostoc mesenteroides	Dark	-	4.88
Leuconostoc pseudomesenteroides	Dark	-	4.99
Leuconostoc pseudomesenteroides	White	+	5.21
Leuconostoc pseudomesenteroides	Dark	-	6.37
Leuconostoc pseudomesenteroides	Dark	-	5.45
Leuconostoc pseudomesenteroides	White	-	4.91
Leuconostoc pseudomesenteroides	White	+	4.91
Leuconostoc pseudomesenteroides	Dark	-	4.99
Leuconostoc pseudomesenteroides	Dark	-	5.21
Leuconostoc pseudomesenteroides	Dark	+	4.99
Leuconostoc pseudomesenteroides	White	-	5.12
Levilactobacillus brevis	White	-	6.48
Limosilactobacillus fermentum	White	-	5.49
Pediococcus acidilactici	White	+	5.88
Pediococcus acidilactici	Dark	+	6.42
Pediococcus acidilactici	Dark	-	6.24
Pediococcus pentosaceus	Dark	+	6.43
Pediococcus pentosaceus	Dark	+	6.43
Pediococcus pentosaceus	Dark	-	6.43
Pediococcus pentosaceus	White	+	6.44
Pediococcus pentosaceus	Dark	-	6.16
Pediococcus pentosaceus	Dark	-	6.52
Pediococcus pentosaceus	Dark	+	5.35
Pediococcus pentosaceus	Dark	-	6.54
Pediococcus pentosaceus	White	-	4.88
Pediococcus pentosaceus	White	+	5.87
Pediococcus pentosaceus	Dark	-	6.24
Pediococcus pentosaceus	White	+	5.03
Pediococcus pentosaceus	White	+	5.12
Pediococcus pentosaceus	White	+	6.43
Pediococcus pentosaceus	Dark	-	6.48

Pediococcus pentosaceus	Dark	+	6.31
Pediococcus pentosaceus	Dark	-	5.35
Pediococcus pentosaceus	Dark	-	5.31
Pediococcus pentosaceus	Dark	+	6.31
Pediococcus sp	Dark	+	6.43

Note: "-" Stands for VP negative results and "+" stands for VP positive results

Table S3. Strains tested for ZEA and OTA removal

NFICC No.	Species
80	Leuconostoc lactis
885	Carnobacterium divergens
314	Leuconostoc pseudomesenteroides
4131	Pediococcus pentosaceus
323	Leuconostoc pseudomesenteroides
4132	Pediococcus pentosaceus
4142	Latilactobacillus curvatus
310	Leuconostoc mesenteroides
825	Leuconostoc mesenteroides
4136	Pediococcus pentosaceus
776	Limosilactobacillus fermentum
852	Pediococcus pentosaceus
234	Latilactobacillus curvatus
4119	Leuconostoc citreum
197	Leuconostoc mesenteroides
1244	Pediococcus pentosaceus
27	Lactiplantibacillus plantarum
72	Lactiplantibacillus plantarum
4146	Lactiplantibacillus plantarum
289	Lactiplantibacillus plantarum
188	Lactococcus lactis
629	Lactiplantibacillus plantarum
862	Lactiplantibacillus plantarum
201	Lactococcus lactis
901	Lactiplantibacillus plantarum
211	Lactococcus lactis
4147	Latilactobacillus sakei
1323	Lactiplantibacillus plantarum
848	Lactococcus lactis

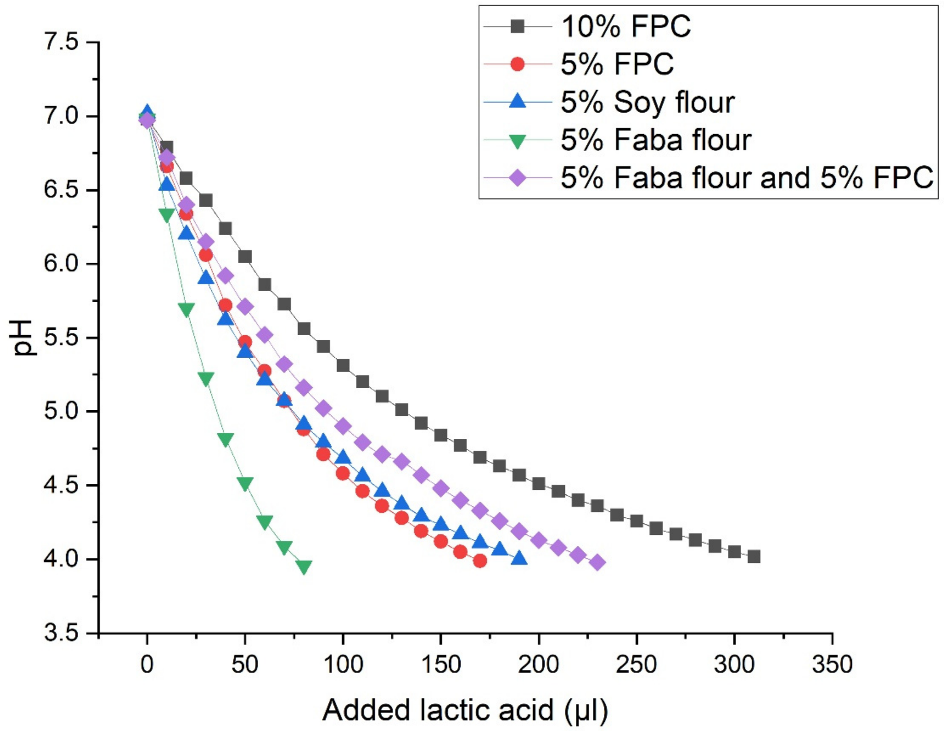

Figure S1. Comparison of the buffering capacity of different plant-based matrices. The buffering capacity of each plant-based matrix was measured using 40 mL of sample titrated with 85% lactic acid.

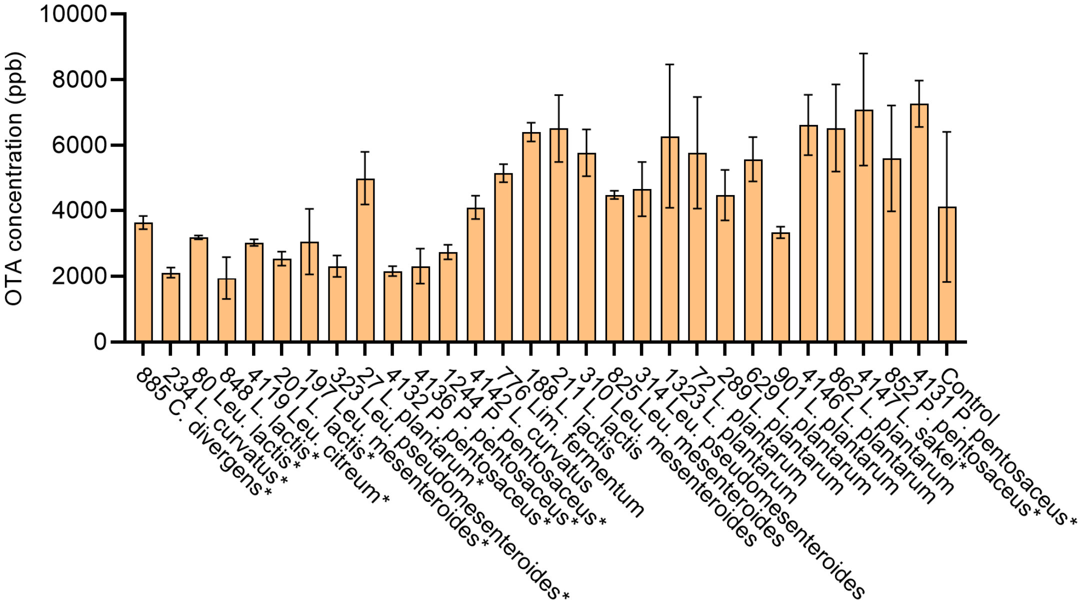

Figure S2: OTA concentrations in supernatant after incubation with various microorganisms. The data represent the analytical duplicates of biological duplicates, analyzed by ELISA assay. Strains marked with an asterisk represent the strains that showed either significantly higher or lower values than the control and were selected for further LC-MS based screening.

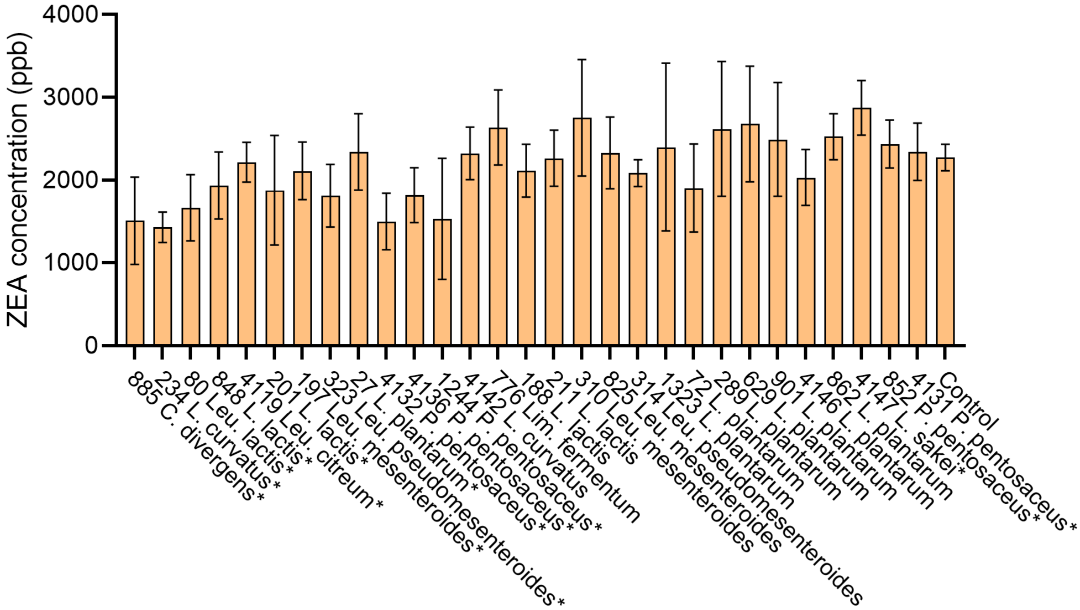

Figure S3: ZEA concentrations in supernatant after incubation with various microorganisms. The data represent the analytical duplicates of biological duplicates, analyzed by ELISA assay. Strains marked with an asterisk represent the strains that showed either significantly higher or lower values than the control and were selected for further LC-MS based screening.

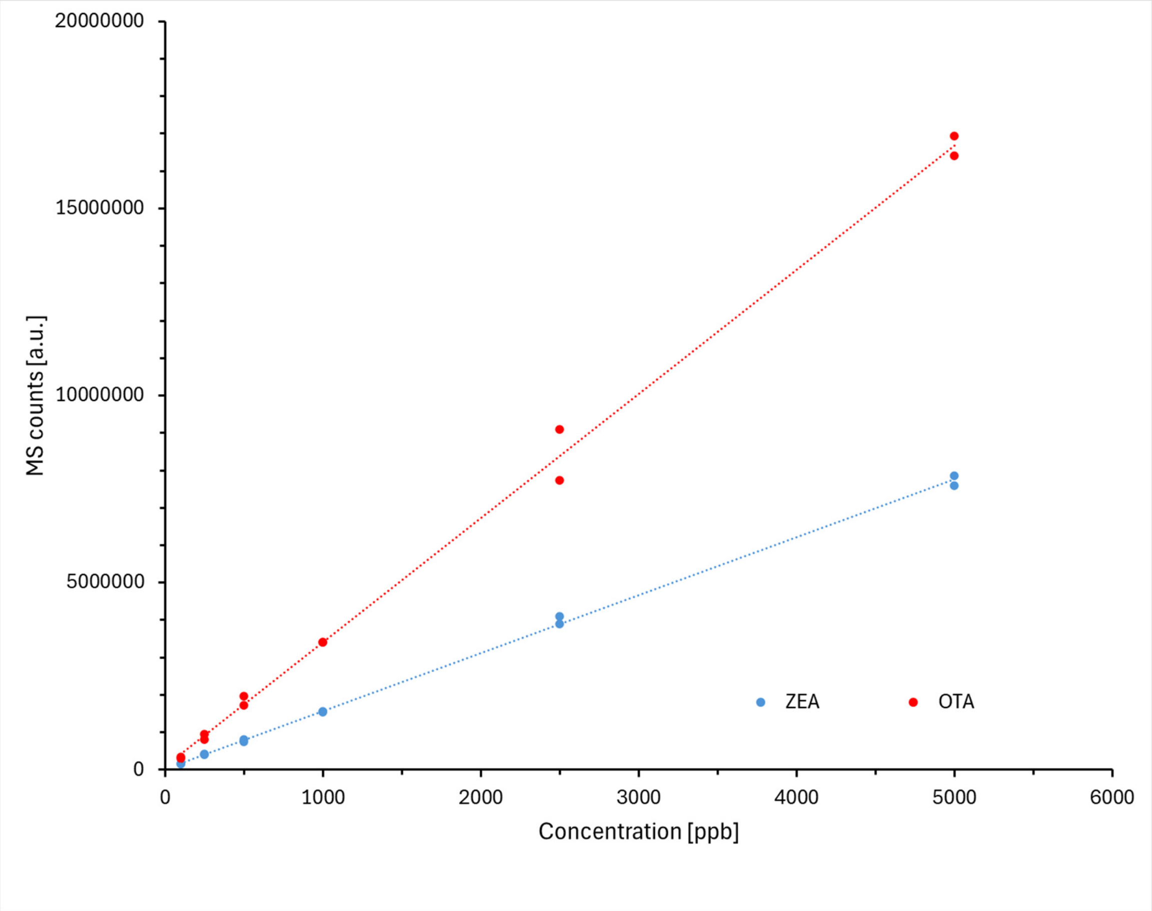

Figure S4: LC-MS standard curve for LC-MS based quantification of ZEA (blue) and OTA (red). Standard concentrations were recorded in duplicates.

Figure S5 (next page): LC-MS chromatogram of supernatant from control incubation of faba bean medium spiked with ZEA and OTA. The upper panel shows the base peak chromatogram (black) and the extracted ion chromatograms for ZEA(magenta) and OTA (cyan). The lower panels show the mass spectra extracted at the retention times of the two compounds. Theoretical m/z values are: ZEA: $[M+H]^+$: 319.154, $[M+Na]^+$: 341.1359, $[M-H_2O+H]^+$: 301.1434 and $[M-2\ H_2O+H]^+$: 283.1329; OTA: $[M+H]^+$: 404.0895, $[M+Na]^+$: 426.0715 and $[M-CO_2+H]^+$ 358.0846 for the ^{35}Cl isotopomers. For the calculation of the extracted ion chromatograms, the m/z values of the ^{37}Cl isotopomers of these three species was also included.

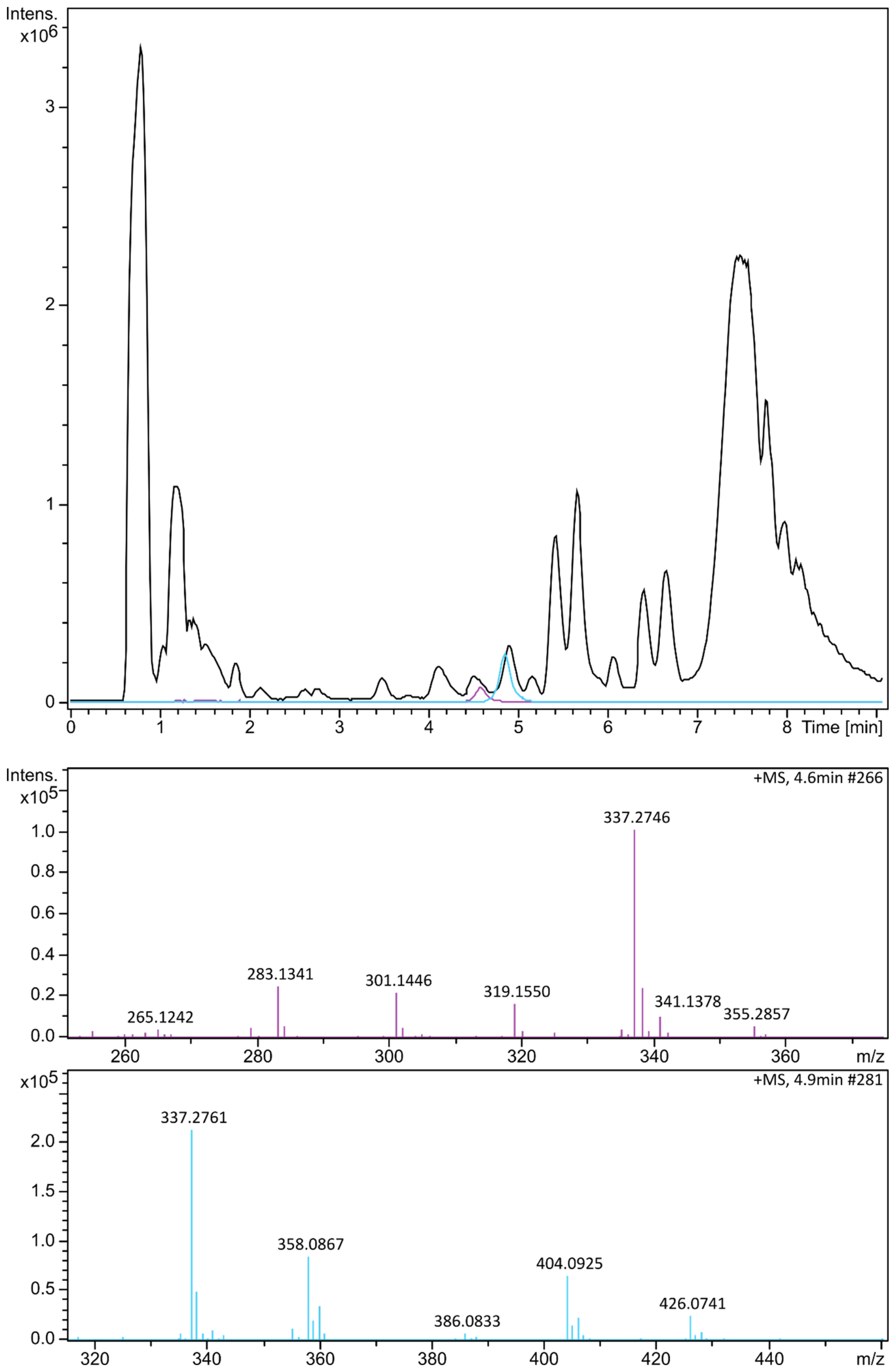
Intens.
x10^6
3
2
1
0
0 1 2 3 4 5 6 7 8 Time [min]
Intens.
x10^5
+MS, 4.6min #266
1.0
0.8
0.6
0.4
0.2
0.0
337.2746
283.1341
301.1446
319.1550
341.1378
355.2857
265.1242
260 280 300 320 340 360 m/z
x10^5
+MS, 4.9min #281
2.0
1.5
1.0
0.5
0.0
337.2761
358.0867
404.0925
426.0741
386.0833
320 340 360 380 400 420 440 m/z
